# Taking it to the extreme: prevalence and nature of extremist sentiment in games

**DOI:** 10.3389/fpsyg.2024.1410620

**Published:** 2024-08-16

**Authors:** Rachel Kowert, Elizabeth Kilmer, Alex Newhouse

**Affiliations:** ^1^Take This, Seattle, WA, United States; ^2^Middlebury College, Center on Terrorism, Extremism, and Counterterrorism, Middlebury, VT, United States

**Keywords:** video games, extremism, terrorism, gaming, trust and safety, toxicity

## Abstract

More than half of all game players report experiencing some form of hate, harassment or abuse within gaming spaces. While prevalence assessments of these actions in digital gaming spaces are ongoing, little remains known about the more extreme forms of these behaviors. Specifically, experiences of extremism. This paper addresses the gap in research knowledge around the expression of extremist sentiment in games by evaluating their prevalence, location, and nature, and impact. Assessing experiences via an online survey, game players (*n* = 423) reported an alarmingly high rate of frequency for being the direct target of, as well as a witness to, all forms of extremist content. Most of these experiences were text-based, reported to be happening in-game. Most players endorsed statements relating to a normalization of extreme ideologies within gaming cultures. It is promising that reporting these behaviors was the primary action taken by players for most of the players; however, “ignoring” these actions was also a common strategy. It is possible that player inaction reflects the embeddedness and normalization of these actions in gaming spaces and/or a lack of trust in moderation systems to be responsive. The prevalence of extreme sentiment in gaming cultures should raise concern from game makers, members of the gaming community, parents, and policy makers alike.

## Introduction

Over half of all game players have experienced abuse in games, with nearly a third (28%) reporting that they experienced it regularly (McBean and Martin, 2020). In 2022, the Anti-Defamation League reported that four out of five adults (86%) reported experiencing harassment in online multiplayer games, representing over 67 million adult gamers. The ADL also found that harassment is increasing among those in the gaming community, with 77% of adults having experienced severe harassment in 2022 (defined as physical threats, stalking, and/or sustained harassment), up from 65% in 2019. Prevalence rates of witnessing abuse in digital games are closer to eight out of 10 of all game players (85.9%; Kowert and Cook, 2022).

It is unclear why harmful actions are so prevalent in gaming spaces. One contributing factor may be the online disinhibition effect (Suler, 2004). When individuals are online they have a sense of anonymity (“*You do not know me*”) and invisibility (“*You do not see me*”) that can reduce their inhibitions to use inciting language. The presence of “toxic gamer cultures” have also been discussed in the academic literature for the last decade (Consalvo, 2012; Busch et al., 2015; Paul, 2018), suggesting a potential cultural element to consider when talking about toxicity in gaming communities. While it is difficult to compare the rates of toxicity across online spaces directly, research has found that individuals perceive gaming spaces as more toxic than social media (Cook et al., 2023). These differences suggest that the toxicity in gaming communities may be unique in the landscape of digital spaces.

Within discussions around toxicity in games, there has been a general failure to differentiate between the different types of hateful or “toxic” behaviors occurring in games. While prevalence assessments, such as the ones noted above, have been helpful in elucidating the prevalence of toxicity, hate, and dark participation more broadly, most have failed to assess the rates of extremist behaviors separate from general hate and toxicity. Extremist rhetoric is a distinct phenomenon from traditional forms of toxicity. However, the expression of “toxic” behavior is, in many cases, a tactic of or gateway into extremist movements (Kowert and Newhouse, 2022, 2023; Braddock et al., 2024).

Extremism is the advocacy of extreme views, including the belief that an in-group’s success or survival can never be separated from the need for hostile action against an out-group (Berger, 2018). Extremist ideology can take many different forms, including the hatred of women (e.g., misogyny) or a particular ethnic group (e.g., racism) or be a more specific set of beliefs and ideologies around a collection of ideas (e.g., White Nationalism, Islamism). There has been a rise in concern regarding extremist sentiments in games for the last several years, by policy makers (Schlegel, 2021; Good, 2022) and the public at large (Kamenetz, 2018; D’Anastasio, 2021). As a growing field of interest, there have been strides in recent years in understanding the using of gaming iconography in propaganda (Schlegel, 2020; Kingdon, 2024), the role of gamification of offline terrorist attacks (Lakhani and Wiedlitzka, 2023), and the organization and mobilization of designated terror groups in these spaces (Ruskin, 2014; Newhouse and Kowert, 2024). For example, a 2021 report from the Institute for Strategic Dialogue (ISD) found significant evidence of extremist communities in gaming and adjacent spaces (i.e., third-party chat servers and streaming platforms). Specifically, they found *Steam* (an online gaming platform) houses a diverse range of public servers created for far-right political parties and violent neo-Nazi groups. In 2019, the ADL did the first known assessment of the prevalence of extremist rhetoric in gaming spaces, finding 23% of online game players are exposed to discussions about white supremist ideology within game spaces. Apart from this work, little remains known about the prevalence or nature of extremist sentiment in gaming spaces.

The aim of this paper is to address the gaps in our research knowledge and growing concern around the expression of extremist sentiment in games by evaluating the prevalence and nature of extremist sentiment in gaming spaces.

## Materials and methods

Participants were asked to complete an online survey and were treated in accordance with ethical and IRB guidelines from Middlebury University. Data were collected for 6 weeks in Q1 of 2023.

### Demographic information

Participants were asked to report their age, gender identity, and country of residence. Due to IRB constraints, only participants over the age of 18 residing in North America and the United Kingdom were eligible to participate. English proficiency was also assessed to ensure participants understood the content of the questions within the survey. Participants were also asked about play frequency habits.

### Prevalence and nature of extreme content

Participants were asked to report what, if any, forms of extremist behavior they had directly experienced (as a direct target), witnessed, and/or participated in within gaming spaces. Specifically, questions related to white nationalism, anti-LGBTQIA+ agitation, misogyny, racism, anti-government extremism, and Islamism were included. These concepts, and their definitions, can be found in Table 1. It is important to note that this list is not meant to be an exhaustive list of ideologies but were selected as they have previously been found to be evident in online gaming spaces [Jenson and De Castell, 2013; Ortiz, 2019; Anti-Defamation League (ADL), 2022; Kowert et al., 2022; Koehler et al., 2023; Wells et al., 2024].

**Table 1:** Forms of extremism and their definitions.

	Definition
White Nationalism	The white nationalist movement is a political movement or grouping based in the United States whose members reject mainstream conservative politics and espouse beliefs and policies centered on the ideas of maintaining a white racial and national identity (Southern Poverty Law Center, n.d.; Berger, 2020).
Anti LGBTQIA + agitation	Anti-LGBTQIA+ agitation is the prejudice, discrimination, marginalization, and/or oppression of people who identify as and/or are perceived to be outside the cisgender, heterosexual “norm.” (i.e., gay, lesbian, bisexual, transgender, two-spirit, demisexual, intersex, asexual, or queer/questioning; Squirrell and Davey, 2023)
Misogyny	Misogyny is a hatred of, contempt for, or prejudice against women. It is a form of sexism that is aimed to keep women at a lower social status than men to maintain the societal roles of patriarchy. Examples of misogyny include violence against women, sexual harassment, and social exclusion (Carian et al., 2022; O’Hanlon et al., 2024)
Racism	Racism is prejudice, discrimination, or antagonism directed against a person (or people) based on their membership to a particular racial or ethnic group (Berger, 2018).
Anti-government extremism	Anti-government extremism is a political movement that believes the federal government is tyrannical and propagates conspiracy theories about an illegitimate government of leftist elites seeing a “New World Order”. This movement has been referred to as the “Patriot” movement. In this context, it is referring to the set of right-wing extremist movements and groups that share a conviction that part or all of the United States government has been taken over by a conspiracy and is therefore not legitimate (Jackson, 2020, 2022).
Islamism	Islamism is a broad term commonly used to describe all movements that adopt a politicized version of Islam, including what is commonly referred to as jihadism (Berger, 2018).

To record where participants’ extremist content was being shared, participants were asked to report where they had primarily experienced and/or witnessed these actions: in gaming spaces, gaming adjacent spaces, or both.

### Expression of extreme content

As the expression of extremism in games can occur through verbal advocacy of extreme views as well as behavioral actions such as the creation of hateful iconography within gaming spaces (e.g., swastikas) and role-playing hateful acts (e.g., role-playing a Nazi within a concentration camp style experience), participants were also asked to report the nature of their experienced interactions by choosing one, or more, of the following options: text/speech, use of associated iconography, use of pictures or images, through in-game role play, or other.

### Player response to extreme content

If participants reported experiencing and/or witnessing these actions, they were asked to report how they have responded to these actions choosing one (or more) of the following options: ignore the comment, report or block the person, confront or challenge the comment publicly, share the information with others in my network, and/or contacted support though the game platform or system.

### Normalization of extremism in gaming spaces

To assess the normalization of extremist sentiment within gamer cultures, participants were asked to report their perception of them as being normalized actions within gaming communities. Specifically, they were asked if they believed each of the extreme sentiments were embedded (i.e., fixed firmly and deeply within) gaming cultures (yes/no). They were also asked to report if they believed “toxic” and/or “hateful” behavior generally was seen as a normalized, or culturally justified, experience within gaming cultures (yes/no).

### Procedure

After providing informed consent, participants were asked a series of questions regarding their exposure and response to, and general perceptions of, extremist ideologies in gaming spaces. With each prompt, participants were presented with the definition of the particular ideology in question (e.g., racism, misogyny) and asked “Have you ever directly experienced (as a target) language or behavior associated with [ideology].” If yes, they were presented with a series of additional questions about the nature of these interactions and their response to the content. After answering questions about direct experiences, they were asked the same series of questions for witnessed experiences. The survey concluded with the questions relating to the normalization of extremism in gaming spaces.

## Results

In total, 423 respondents completed the survey. Fifty-six participants had to be removed from the analyses as they did not reside in the United States or United Kingdom. An additional six participants were removed who failed an embedded validity question or showed patterns of data disruption. The final dataset resulted in 361 observations.

Most participants identified as men (49.6%), with women (30.2%) and non-binary gender non-conforming, or questioning (19.7%) constituting most of the rest of the sample. Two participants (0.6%) declined to provide their gender. Participants ranged in age from 18 to 63, with an average age of 33 (SD = 7.71). Most participants resided in the United States (85.3%), while a smaller proportion resided in the United Kingdom (14.7%).

### Prevalence and nature of extremist content

Overall, 86.43% participants reported witnessing and being the direct target of some kind of extremist content. The most frequently witnessed categories were misogyny (76.73%), racism (76.73%), Anti-LGBTQIA+ sentiment (75.90%). For being directly targeted, the most frequently reported was Anti-LGBTQIA+ (49.31%), misogyny (47.09%), and White Nationalism (31.30%). The prevalence of all forms of extremist content can be seen in Table 2.

**Table 2:** Percentages of participants (*n* = 361) who experienced and witnessed extremist sentiment in digital gaming spaces.

	Witnessed	Direct target
Misogyny	76.73	47.09
Racism	76.73	24.38
Anti-LGBTQIA+	75.90	49.31
White Nationalism	61.50	31.30
Anti-Government	29.10	14.40
Islamism	25.21	7.20

Chi-square tests of independence were utilized to examine potential relationships between gender and extremism experiences. These analyses were limited to the gender categories of men and women as there were too few participants (*n* = 71) in the other gender categories.

Women were found to be more likely to be direct targets of misogyny [𝛸^2^(2, *N* = 296) = 113.75, *p* < 0.001]. Men were more likely to be direct targets of racism [𝛸^2^(2, *N* = 296) = 9.87, *p* < 0.01] and Islamism [𝛸^2^(2, *N* = 296) = 6.86, *p* < 0.05]. Men were found to be more likely to witness anti-government sentiment than women [𝛸^2^(2, *N* = 317) = 11.08, *p* < 0.01]. No other significant gender differences were found.

Half of all participants (51.89%) reported witnessing extreme behavior primarily in gaming spaces, more than a third noted they had witnessed extremist rhetoric in game and game-adjacent spaces equally (37.42%), while 10.69% reported only witnessing extremist content in game-adjacent spaces. Among those who had been directly targeted by extremist sentiment, nearly three-fourths (72.39%) of participants reported this happening in gaming spaces, 21.89% in game and game adjacent spaces equally, and only 5.72% reported being directly targeted only in game adjacent spaces.

### Expression of extremist content

#### Misogyny

Most direct (99.41%) and witnessed (100%) experiences of misogyny were experienced via text and speech channels. The use of iconography (31.18% direct target, 40.07% witness), pictures or images (41.76% direct target, 48.74% witnessed) and in-game role play (45.29% direct target, 42.29% witnessed) were also evident.

#### Racism

Most direct (97.73%) and witnessed (99.64) experiences of racism occurred via text and speech channels. However, the use of iconography (42.05% direct target, 51.99% witness) and pictures or images (36.36% direct target, 52.35% witness) were also common. Role-playing was the least common way to experience racism in gaming spaces (26.14% direct target, 36.10% witnessed).

#### White nationalism

Most direct (90.27%) and indirect (95.95%) experiences of white nationalism were experienced via text and speech channels. However, iconography (62.83% direct target, 68.47% witness), pictures and images (52.21% direct target, 52.70% witness) and in-game role play (22.12% direct target, 27.48% witness) were also reported.

#### Anti-LGBTQIA+ agitation

Most direct (99.44%) and indirect (98.91%) experiences of Anti-LGBTQIA+ agitation was experienced via text and speech channels. Iconography (26.97% direct target, 37.59% witnessed), pictures and images (27.53% direct target, 36.50% witness), and in-game role play (27.53% direct target, 29.93%, witness) were also reported.

#### Anti-government sentiment

Most direct (96.15%) and witnessed (99.05%) experiences of anti-government sentiment were through text and speech channels. Iconography (38.46% direct target, 53.33% witnessed), pictures or images (46.15% direct target, 52.38% witnessed) and in-game role play (25.00% direct target, 23.81%, witnessed) were less frequently reported.

#### Islamism

Most direct (92.31%) and witnessed (98.90%) experiences of islamism were through text and speech channels. Nearly half of all participants also reported directly experiencing (46.15%) and witnessing (49.45%) Islamism through the use of iconography. Pictures or images (30.77% direct target, 48.35% witnessed) and in-game role play (15.38% direct target, 24.18% witnessed) were frequently reported.

### Player response to extremist content

Across categories the most common response by players was to report or block, and ignoring the comment was the second most reported strategy. Less frequently utilized strategies included challenging the comment publicly, sharing information with others in the network, and contacting in-game or in-platform support (beyond the common reporting features). The most common strategies utilized by direct targets can be found in Table 3. The most common strategies utilized by witnesses can be found in Table 4.

**Table 3:** Reported player strategies as a direct target of extremist sentiment.

Player strategy	Misogyny	Racism	White Nationalism	Anti-LGBTQIA+	Anti-Government	Islamism
Report/block	86.47	87.50	90.27	90.45	69.23	65.38
Ignore	80.00	75.00	60.18	71.35	80.77	92.31
Confront publicly	49.41	43.18	29.20	0	36.54	19.23
Share information	37.65	21.59	32.74	34.27	25.00	11.54
Contact support	42.35	37.50	51.33	39.33	28.85	19.23

**Table 4:** Reported player strategies as a witness of extremist sentiment.

Player strategy	Misogyny	Racism	White Nationalism	Anti-LGBTQIA+	Anti-Government	Islamism
Report/block	88.45	90.61	90.99	91.61	74.29	80.22
Ignore	73.29	66.79	63.51	70.07	78.10	76.92
Confront publicly	51.62	46.93	36.04	47.45	32.38	37.36
Share information	32.13	31.77	29.28	29.93	32.38	36.26
Contact support	38.99	42.96	45.05	41.97	40.95	27.47

### Normalization of extremism in gaming spaces

Most participants agreed that misogyny (76.45%), racism (63.71%), and Anti LGBTQIA+ agitation (59.56%), have become embedded or normalized in gaming cultures. A substantial proportion of participants agreed White Nationalism (41%), Islamism (19.11%), and anti-government sentiment (19.11%) have also become normalized within digital gaming cultures.

Notably, women were more likely than men to report beliefs that white nationalism [𝛸^2^(1, *N* = 288) = 9.26, *p* = 0.002], anti-LGBTQIA+ agitation [𝛸^2^(1, *N* = 288) = 12.30, *p* < 0.001], misogyny [𝛸^2^(1, *N* = 288) = 19.81, *p* < 0.001], racism [𝛸^2^(1, *N* = 288) = 11.74, *p* < 0.001], and Islamism [𝛸^2^(1, *N* = 288) = 5.54, *p* = 0.019] are embedded or normalized in gaming cultures.

## Discussion

Dark participation in games is a topic of active discussion among scholars and the public alike. There is great concern over the presence, prevalence, and normalization of hate, harassment, and other harmful behaviors within our digital playgrounds. However, when it comes to discussing dark participation in games, it is important to understand that not all behaviors are created equal. This paper assessed the prevalence and nature of extreme forms of dark participation by analyzing the prevalence of direct and indirect exposure to extreme ideologies within gaming spaces. Results show an alarmingly high rate of frequency for being the direct target of, as well as a witness to, all forms of extremist content: misogyny, racism, anti-LGBTQIA+ sentiment, White Nationalism, anti-government sentiment, and Islamism.

Most of the incidents were reported to be text-based and in-game. This finding may be related to greater psychological detachment present in text-based communication via the online disinhibition effect (Suler, 2004). That is, the sense of invisibility and anonymity may be heightened when using text-based, versus voice based chat, leading individuals to feel more emboldened to share hateful rhetoric. Regardless of the reason, finding most of the incidents to be in-game and text-based may be the best-possible scenario, as many gaming companies and third-party platforms are already addressing text-based toxicity with their ongoing moderation efforts. However, the sheer prevalence of encounters with extremist behavior indicates a lack of effectiveness of existing tools.

It is promising that reporting these behaviors was the primary action taken by players for most of the players. This supports previous work that has found most people actively report toxic behaviors in gaming spaces (Kowert and Cook, 2022). However, it is notable how common “ignoring” these actions are. This could point to the ubiquity and normalization of extremist content in gaming spaces as well as a general lack of trust in moderation systems (which has been noted in previous research, see Cook et al., 2018; Kowert and Cook, 2022). Notably, anti-government sentiment is the only extreme sentiment where the primary strategy was to ignore, both as a direct target and a witness. It is unclear why this is the case, but it may be because anti-government rhetoric may be less likely to be language directed at another player directly. Further research is needed to better understand why these behaviors are not actioned by players. It is also worth highlighting that not a single direct target of Anti-LGBTQIA+ agitation reported that they confronted these behaviors publicly. This may reflect a fear of retaliation or a lack of faith in the effectiveness of existing reporting systems.

Perhaps the most alarming finding is the normalization of extreme behaviors within gaming communities. All forms of extreme behavior were found to be a relatively commonplace experience, with misogyny, racism, and anti-LGBTQIA+ agitation being noted as more the norm than the exception. White Nationalism, anti-government sentiment, and Islamism were also witnessed by more than one in five of all players. The sheer prevalence of these findings should raise concern from game makers, members of the gaming community, parents, and policy makers alike.

Notably, female players were more likely to note the normalization of most forms of extremist rhetoric. While it is unclear why this is the case, it is possible that the marginalization of women in gaming spaces (Gray et al., 2017) has contributed to a heightened awareness of extreme behaviors, particularly as they often report a lack of agency to push back or change the hostile norms they observe (Drury and Kaiser, 2014). Further research is needed to explore this possibility.

Moving forward, it is imperative to consider extremist sentiment when discussing “toxicity” in games. As even mere exposure to extremist ideologies can have significant interpersonal and societal consequences, future policy and intervention work should focus on prioritizing their efforts to reduce extremist language in gaming spaces. Individuals adjust to the norms of their culture (even across digital spaces; Smith et al., 2021; Kowert et al., 2022).The normalization, and cultural embeddedness, of extremist language in games puts players are at risk of being socialized into hateful and extreme ideologies from within their own groups as well as through contagion from the broader gaming community (Sooknanan and Comissiong, 2017). The sheer prevalence of this language also makes gaming spaces more vulnerable to more direct forms of extremist exploitation, such as propaganda dissemination (Kingdon, 2024) and recruitment efforts (Koehler et al., 2023; Kowert and Newhouse, 2023). The adoption of extreme beliefs online is often discussed as the beginning of a radicalization pipeline that begins with the normalization of extremist rhetoric, grows into to acclimation, and ends with the dehumanization of others, which is the foundation for escalated action and an important psychological prerequisite of violence (Munn, 2019).

Industry efforts should reevaluate their policies to ensure they are addressing these particular vulnerabilities. While the kinds of tools and strategies will likely be tailored to each particular studio or community, there are commonalities across spaces making industry-wide conversations about these topics are vital (Kilmer and Kowert, 2024). For example, while text-based harm was the most common modality reported here, text-based moderation cannot be the only solution. A significant number of players also reported experiencing and witnessing extremist rhetoric through the use of iconography, pictures, and images. It will be important to consider these other modalities when developing new policies and moderation tools.

### Limitations

While this work provides insight into the prevalence of extremist sentiments in digital gaming spaces there are several limitations to consider. First, it only evaluated five types of extremist ideologies. While these were selected as they have already been documented within digital spaces, it is possible that it does fully encapsulate the range of extremist ideologies being propagated in gaming spaces. Another limitation of this study is grouping “report” and “block” as a single answer when asking participants for their response to the action. However, as contacting in-game or platform support was also an option, it is possible to infer that the choice of “report/block” nearly always reflects utilizing the block feature (perhaps, but not always in addition to reporting). Blocking is a self-preservation strategy but may not effectively curb the culture or action of the “bad actor” within the gaming space. Additional research is needed to tease out the frequency with which individuals are acting to self-preserve (through blocking) as compared to culture change (through reporting). The authors hypothesize that the former is more common than the latter judging by the discrepancy between rates of “report/block” as compared to “contacted in-game support.” This is important as many companies rely on player reports as their primary strategy of reactive moderation. Lastly, this work drew from an opportunity sample of participants willing to share their thoughts on dark participation in games. Representative sampling would help elucidate some of the potential biases an opportunity sample may bring, such as a potential overrepresentation of these experiences.

## Conclusion

The propagation of extremist ideologies in gaming spaces is multi-faceted and includes the development of bespoke games, the creation of propaganda, as well as social influencing (Wells et al., 2024). We also know that the opportunity for these behaviors to propagate in gaming spaces is unique as compared to other spaces on the internet (Kowert and Newhouse, 2022), due to a lack of effective moderation (Makuch, 2019; Davey, 2021), the high prevalence of “toxic” behavior within these spaces [McBean and Martin, 2020; Anti-Defamation League (ADL), 2022], as well as the unique social features of games themselves (Kowert and Newhouse, 2022). Despite this, there has remained a dearth of knowledge regarding the kinds of ideologies that are being propagated and normalized within these spaces. This study brings some needed clarity to the landscape of extremist sentiment in games, illustrating the prevalence, nature, and scope of extremist rhetoric in game (and game adjacent) platforms. The results of this work confirm what has long been feared: the ubiquity of, and cultural normalization, of extremist rhetoric and behavior within gaming spaces. Moving forward, it is important to consider extremist sentiments as part of the landscape of so-called “toxic” behavior when developing moderation and resiliency efforts within gaming and game-adjacent spaces (Kilmer and Kowert, 2024). However, it is important to clarify that these findings do not suggest that gaming *causes* radicalization or extremism. Rather, extremist language is commonplace in gaming spaces. Such consistent exposure is cause for considerable concern in and of itself and may be setting the foundation for direct extremist exploitation of our digital playgrounds.

## Data availability statement

The raw data supporting the conclusions of this article will be made available by the authors, without undue reservation.

## Ethics statement

The studies involving humans were approved by Middlebury Institute of International Studies. The studies were conducted in accordance with the local legislation and institutional requirements. The participants provided their written informed consent to participate in this study.

## Author contributions

RK: Conceptualization, Data curation, Formal analysis, Funding acquisition, Investigation, Methodology, Supervision, Writing – original draft, Writing – review & editing. EK: Data curation, Formal analysis, Investigation, Methodology, Writing – review & editing. AN: Conceptualization, Funding acquisition, Writing – review & editing.
